# Patient narratives in an online hernia peer-support community: a qualitative netnographic analysis

**DOI:** 10.1007/s10029-026-03860-y

**Published:** 2026-09-25

**Authors:** Asim A. Abbas, Natyra Xharavina, Jackie Bullock, Srinivas Chintapatla

**Affiliations:** 1 York Abdominal Wall Unit, Department of General Surgery, York and Scarborough Teaching Hospitals NHSFT, 4th Floor, Administrative Block, Wigginton Road, Clifton, York, YO31 8HE UK; 2 Department of Management and Marketing, University of Westminster, London, UK; 3 Patient Representative, London, UK

**Keywords:** Hernia, Netnography, Qualitative, Online health information seeking (OHIS), Health-related quality of life (HRQoL)

## Abstract

**Purpose:**

This study aimed to explore how individuals with hernia engage with online peer-support communities and how their shared narratives reflect the experience of living with and seeking treatment for hernia.

**Methods:**

A qualitative netnographic approach was employed, analysing posts from a large online hernia support group with approximately 8,500 members across more than 10 countries. The group was selected following scoping of alternative online communities because of its size, activity, heterogeneity, and public accessibility. A purposive sample of 26 high-engagement discussion threads comprising 936 comments was analysed using Braun and Clarke’s reflexive thematic analysis, involving iterative familiarisation, inductive coding, theme development, and refinement. Saturation was monitored throughout, and data collection concluded when no new dominant themes were identified.

**Results:**

Four inductively derived themes were identified: Surgical Experiences and Outcomes, Patient Concerns and Fears, Healthcare System and Access, and Coping and Support. Narratives described mesh-related anxiety, fear of recurrence, mistrust in healthcare providers, structural barriers to care, body-image concerns, and uncertainty surrounding treatment and recovery. Mesh discussions included concerns about safety, long-term complications, and living with an implanted material, alongside positive experiences of mesh repair. Participants also demonstrated active health-information-seeking behaviours, including peer validation, appraisal of surgical options, and self-advocacy. A visual summary of findings is included (see accompanying infographic).

**Conclusion:**

Online patient narratives offer rich insight into the lived experience of hernia and demonstrate how peer communities contribute to information seeking, support, and treatment-related discussion. These exploratory findings identify issues relevant to patient-centred communication and future outcomes research and suggest value in recognising the digital patient as an active information-seeker and co-producer of meaning.

## Introduction

Hernias are among the most common conditions encountered in general surgery, with significant variation in symptom burden, treatment pathways, conservative and operative management choices, and long-term outcomes [1]. Patients with complex or recurrent hernias frequently experience protracted and distressing symptoms that have a significant impact on quality of life (QoL) [2, 3]. There is also a specific impact on body image [4], mental health [5], employment, social relationships [6], and function [7]. This disruption to QoL can lead to patients seeking support and guidance beyond the clinical setting [8].

In recent years, social media and online peer support communities have become increasingly important sources of patient driven information and advocacy [9–11]. These platforms offer spaces for individuals to share personal experiences, exchange advice and information, and provide mutual encouragement. For patients with hernia, these groups may represent an avenue for support and navigating uncertainty. However, while the use of online forums in other areas of healthcare, such as mental health [12, 13] or cancer services [14], has been well documented, little is known about how patients with hernias use these digital spaces, what narratives they construct, and how this engagement might shape their perceptions of their condition, their expectations of care, and their interpretation of formal health interactions such as surgery or quality of life assessment [15, 16].

This study aimed to explore the role of online peer support in the lived experience of patients with hernia through a netnographic analysis of a large, publicly accessible Facebook support group with over 8,000 members. Netnography is a qualitative method adapted from ethnography for the study of online communities [17]. It involves the systematic observation and thematic analysis of naturally occurring digital interactions to uncover the beliefs, values, and behaviours shared within virtual environments. In this context, netnography offers a unique opportunity to explore how patients conceptualise their hernia experiences outside clinical settings, when the surgeon is “out of the room”. It may also allow examination of how informal peer support intersects with or diverges from professional medical guidance.

Understanding these dynamics is particularly relevant in the context of development of patient reported outcome measures (PROMs) and health related quality of life (HRQoL) tools in AWH. As the development and implementation of AWH-specific HRQoLs evolve, it is important to consider how patients interpret these tools in relation to their lived experiences and the social narratives they engage with. Patients are increasingly interacting with each other in online communities and support groups, and it is felt that these interactions should be ethically studied to understand the themes being discussed. This study therefore aims to map the dominant themes of narratives and interactions within peer support forums. Any comparison with existing AWH quality-of-life concepts was exploratory and was not intended to validate or revise an existing model or PROM.

## Methods

### Study design

This study employed a qualitative, netnographic design to explore how individuals with hernia engage with other users of an online peer support community. Netnography, a form of ethnography, explores naturally occurring interactions in digital communities, including online forums. Originally used as a research tool in marketing, netnography offers insight into the lived experiences, shared narratives, and collective meaning-making of patients outside traditional healthcare environments [18, 19]. Netnography was first described by Kozinets in 2002 [20], and later updated in 2024 by Kozinets and Gretzel, where six stages are described: Initiation, Immersion, Investigation, Interaction, Integration, and Incarnation [21]. This study employed all except “Interaction” as a passive stance was selected, with no active interaction between the research team and community participants, so as not to influence organic user-user interactions.

This study adopts a constructivist epistemology, recognising patient narratives as situated, co-constructed accounts of lived experience. Drawing on the interpretivist tradition, netnography enables exploration of meaning-making in digitally mediated peer interactions, acknowledging the legitimacy of online discourse as a source of experiential knowledge [20, 22].

### Ethics

Ethics was sought and granted by Hull York Medical School (HYMS), with ethics reference HYMS-25-26-003. Given the internet‑mediated nature of this research, we adhered to established guidance on ethical conduct in online contexts [23], noting contemporary literature highlights that internet‑based research can pose distinct ethical challenges [24, 25].

### Setting and data source

The data source for this study was a large, publicly accessible Facebook support group dedicated to individuals affected by hernia (“Facebook Hernia Support Group”; URL: [https://www.facebook.com/groups/262467366015030/*]).* At the time of data collection, the group comprised approximately 8,500 members, of whom approximately 5,000 were defined by Facebook as active users. The group was launched in 2022, following an open forum between patients and surgical colleagues. The group is moderated by a small team, including a patient representative (JB), who is a collaborator on this project to ensure transparency, sensitivity, member-checking, and honest handling of community generated content. The group operates independently as a patient-led peer-support community and was not established for legal, commercial, or industry purposes. Although moderated, no evidence of systematic exclusion of viewpoints or overt agenda-driven content was identified during data collection.

At the time of data collection and analysis, the group’s membership displayed a broad international spread, with the largest proportions from the United States (3,260), United Kingdom (1,955), and the Philippines (558), alongside active members from Australia, South Africa, Canada, and several countries across Africa and Asia. Table 1 shows a breakdown by geographic spread showing the ten most frequent countries of group members. This suggests a degree of global reach and cultural heterogeneity, although the discourse remained largely English-speaking. The gender distribution was skewed towards female users (70% female vs. 30% male), with the most active age groups being 35–44 and 45–54 years. Discussion topics reflected a spectrum of hernia experiences, including incisional, ventral, umbilical, inguinal, and complex abdominal wall reconstruction narratives. These analytics were used to contextualise the community rather than to characterise individual participants.

The group was not restricted to complex abdominal wall reconstruction and did not apply a formal clinical definition of hernia type or operative complexity. Rather, it functioned as a broad peer-support community for individuals affected by hernias, with posts reflecting a spectrum of hernia experiences. Where identifiable from the content, discussions included umbilical, incisional, ventral, inguinal, parastomal, recurrent, and complex abdominal wall hernia narratives. However, hernia type and operative technique were not consistently reported by posters and could not be reliably classified for every thread.

**Table 1:** Geographic breakdown of group membership, as of December 2025

Country	Number of members (*n*)	Percentage of group (%)
United States	3,260	38.7
United Kingdom	1,955	23.2
Philippines	558	6.6
Australia	395	4.7
South Africa	334	4.0
Canada	285	3.4
Nigeria	198	2.4
Kenya	141	1.7
India	111	1.3
Ireland	85	1.0

Several other potential online data sources were initially considered. These included a group focussed on hernia mesh complications on Facebook (approximately 1,200 members), and a global “hernia” blog and discussion forum (which, at the time of scoping, had a relatively low level of recent activity, with only 12 discussion threads posted in the preceding two months), and the large public Facebook support group ultimately selected. The former, while active and patient-led, focuses narrowly on mesh-related concerns and attracts a highly specific subset of patients whose experiences may not be representative of the broader hernia population. As such, analysis of this group alone risked emphasising only complication-focused narratives and limiting the diversity of perspectives captured. The latter, although valuable as a specialist resource, contained substantially fewer discussions and offered a less interactive environment, with posts tending to be shorter, less conversational, and less reflective of peer-to-peer emotional or decisional support.

In contrast, the selected Facebook group provides a large, heterogeneous, and highly active community, offering a wider spectrum of patient experiences across the full trajectory of hernia. Its content is consistently rich, comprising long-form text, iterative comment threads, shared images, and varied peer responses. This multimodal and dialogic nature allows for more nuanced exploration of patient meaning-making. For these reasons, the selected group was judged to be the most appropriate and methodologically robust source for netnographic analysis.

### Data collection

Data collection was multi-staged, in keeping with Kozinets’ descriptions of Netnography [20]. Data collection was conducted through a process of non-participant observation. Posts were identified through a systematic review of group activity over the preceding 18-month period, using Facebook’s internal chronological navigation features. Data extraction was undertaken in December 2025. Eligible posts were identified retrospectively from discussion threads published between June 2024 and November 2025, representing a predefined 18-month sampling window. Restricting analysis to a predefined retrospective period was intended to provide temporal consistency while maintaining sufficient breadth to capture recurring discussion patterns. Only posts publicly visible within the group, with sufficient engagement (defined as > 10 comments) to enable thematic richness were included. Sampling was purposive and sought to maximise diversity of discussion topics and experiences rather than privilege dominant viewpoints or recurrent narratives. Both concordant and discordant perspectives were retained during analysis. A standardised data extraction template was used to capture key features of each thread, including the overall topic, the number and type of responses, and the presence of recurring language, metaphors, or themes relevant to the study aims. To preserve confidentiality, all posts and comments were paraphrased and anonymised. No identifiable information was recorded. A structured data matrix was used to document the paraphrased content alongside key metadata such as post date, topic summary, type of engagement, and number of responses. Each post was inductively coded to identify emerging thematic patterns. Findings were triangulated across the research team.

Importantly, no direct contact was made with group members at any stage of the research. To protect the privacy and dignity of participants, all data was anonymised at the point of extraction, with particular attention given to the risk of indirect identification through searchability or contextual clues. A conscious decision was made not to transcribe posts verbatim. While posts on public platforms may be technically accessible, direct quotations (especially when indexed by search engines) can be traced back to the original author using simple keyword searches. This “digital footprint” can pose a significant risk to user anonymity, even when personal names or profile details are omitted [26]. Individual demographic variables (e.g. age and sex) were not extracted at poster level, as the study was designed around thread-level analysis and demographic inference from public profiles was considered unreliable and potentially ethically problematic.

### Data analysis

Data were analysed using reflexive thematic analysis, guided by Braun and Clarke’s six-phase framework [27, 28]. Reflexive thematic analysis was selected because of its compatibility with interpretivist qualitative inquiry and its suitability for exploring meaning-making within naturally occurring online interactions. Analysis moved iteratively between data familiarisation, coding, theme generation, and interpretation rather than proceeding in a strictly linear sequence.

Following data extraction, researchers repeatedly reviewed paraphrased posts and discussion threads to achieve immersion in the dataset and identify recurrent patterns of meaning. Initial coding was performed inductively at semantic and latent levels, capturing both explicit content and underlying concepts. Codes were developed iteratively and refined through constant comparison across threads.

Related codes were then grouped into candidate themes and subthemes through discussion within the research team. Themes were reviewed against the original dataset to ensure internal coherence and conceptual distinction, before being refined and defined into a final thematic structure. Themes were developed based on conceptual richness and recurrence across the dataset rather than frequency counts. Descriptive frequency language was used cautiously to indicate thematic presence across discussions and should not be interpreted as prevalence estimates. Throughout analysis, reflexive discussions were used to interrogate assumptions and consider alternative interpretations.

Reflexive journaling by the research team was employed to support transparency and positionality throughout the analytic process [29]. The lead researcher is a surgical research fellow with experience in hernia management, which enabled clinical insight but also necessitated ongoing reflexive practice to remain open to patient framings that diverge from medical orthodoxy. Reflexive journaling and peer debriefing were used to mitigate bias during theme generation [30]. Analytic triangulation was undertaken through independent coding and iterative team-based review, with discrepancies discussed and resolved through reflexive dialogue, ensuring credibility and trustworthiness of findings in accordance with SRQR guidance [31]. Particular attention was given to contradictory cases and minority perspectives during theme refinement to minimise overrepresentation of dominant narratives and strengthen interpretive credibility.

## Results

Analysis of 26 discussion threads comprising 936 comments across an 18-month period, revealed four overarching inductive themes, each comprising several interconnected subthemes. These themes were generated inductively from the dataset and are presented below with illustrative paraphrased quotations. They spanned a broad range of hernia-related experiences, from primary and recurrent hernias to complex reconstruction narratives. The themes reflect recurring patterns of meaning across discussions and are presented to illustrate interpretive salience rather than estimate prevalence.

**Fig. 1:**
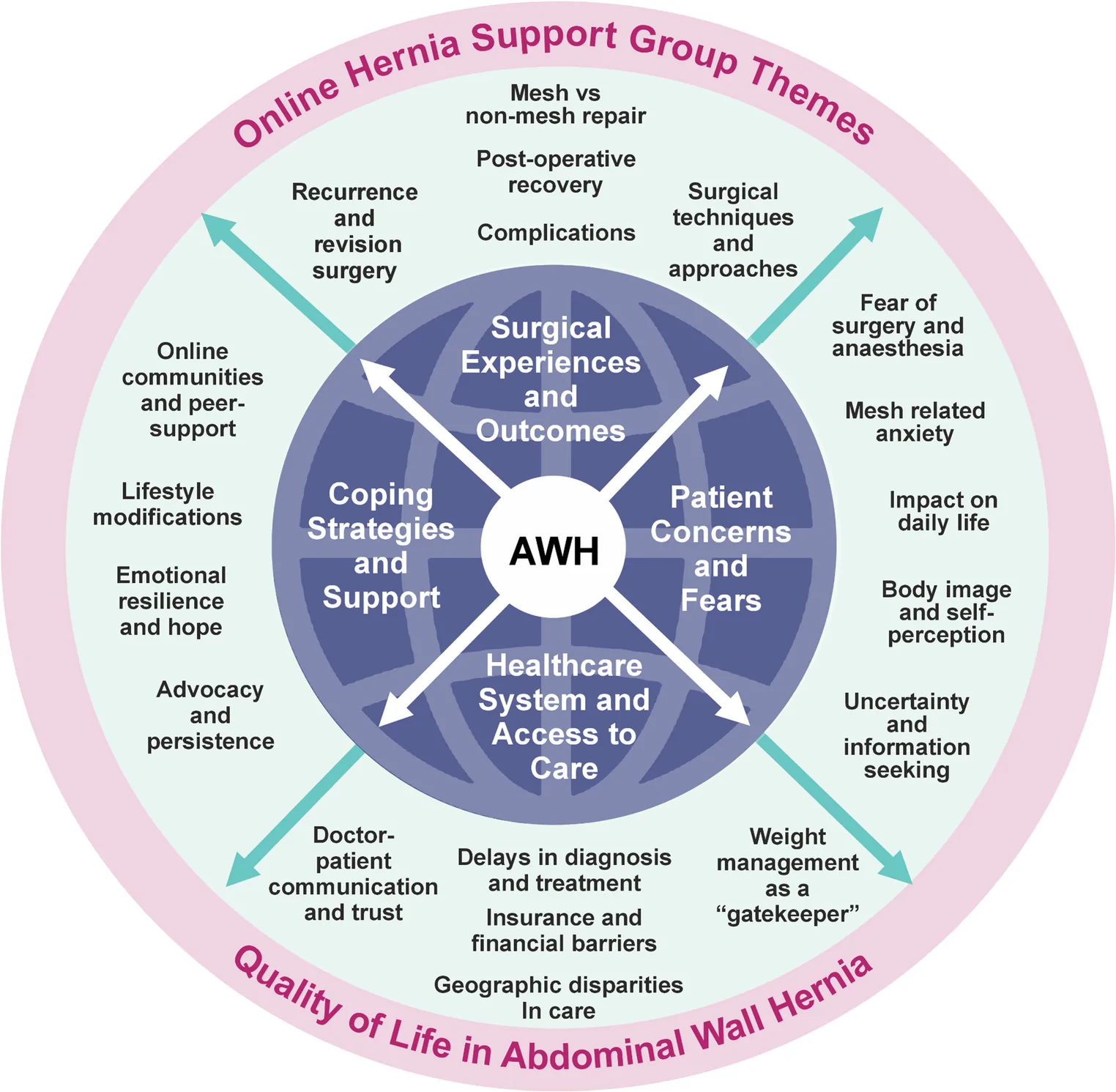
Framework of inductive themes from netnographic analysis of online hernia support group

Figure 1 is a visual representation of the resulting framework illustrating four interconnected thematic domains derived from inductive thematic analysis of a large public Facebook group for hernia patients.

Table 2 provides a summary of the four inductive themes, each illustrated with an indicative paraphrased quotation.

**Table 2:** Summary of inductive themes with indicative paraphrased quotations

Inductive theme	Indicative quote (paraphrased for anonymity)
Surgical experiences and outcomes	*“I’ve had four surgeries for the same hernia. It just keeps coming back. They now say there’s nothing more they can do.”*
Patient concerns and fears	*“Does surgical mesh leech into the body and cause cancer or complications later in life?”*
Healthcare system and access	*“I told my doctor something felt wrong*,* but he kept dismissing it. Only when I ended up in A&E did they finally find the hernia.”*
Coping and support	*“This group saved my sanity. I felt so alone before. Now I know others are going through the same and surviving it.”*

## Theme 1: surgical experiences and outcomes

This major theme captured the diverse range of experiences shared by individuals undergoing or contemplating hernia surgery. Narratives reflected both satisfaction and distress, often shaped by surgical approach, use of mesh, complications, and recovery trajectories.

### Surgical techniques and approaches

Posters discussed laparoscopic, open, and robotic techniques, often in relation to recovery time or recurrence risk. One post compared “*Mayo vs. Spitzy*” techniques, while another noted “*I chose laparoscopic because it seemed less invasive*,* my recovery was faster than expected*.” Others reported that surgeons discovered more hernias intra-operatively than expected, expressing their shock at this unexpected finding, the disease burden, and a loss of confidence in the surgeon.

### Mesh vs. non-mesh repair

Multiple contributors discussed the perceived risks and benefits of mesh. Some described serious complications or symptoms that they attributed to mesh, including systemic symptoms attributed to “*toxins leeching from the material*” or “*mesh rejection*” requiring removal. One contributor described mesh as “attaching to my organs,” which they associated with subsequent emergency surgery. In contrast, others shared success stories: one patient noted, “*I’ve had mesh for years with no issues*,* it gave me my life back*.” Several actively sought out “*no mesh*” options such as the Shouldice technique, citing fears of long-term complications or lawsuits.

### Post-operative recovery

Recovery experiences varied widely. Some described straightforward recoveries: “*I was walking by day three and back to light chores in a week*.” Others experienced severe post-operative pain, prolonged immobility, or unanticipated complications. One user recounted needing help from their children just to get out of bed and feed the pets, stating, “*I underestimated how hard this would be and I couldn’t bend for days*.” Concerns described across discussions included constipation, drain management, and fatigue, alongside strategies such as stool softeners, dietary modification, and pacing.

### Complications

Contributors described a range of complications, including infections, bowel obstruction, and problems attributed to mesh. One user detailed an experience of “*entrapment*,” where bowel loops twisted post-operatively, leading to emergency admission and nasogastric decompression: “*Two days of no food*,* just bile being pumped out*.” Others mentioned wound infections, delayed healing, or seromas. A few described undergoing mesh removal due to chronic pain or erosion.

### Recurrence and revision surgeries

Recurrence emerged as a recurring concern across discussions. Patients described undergoing multiple surgeries: “*This is my fourth repair… it just keeps coming back*.” In some cases, individuals expressed helplessness and frustration, reporting that “*the surgeon said there was nothing more they could do*”. These stories often included speculation about surgical technique or inadequate initial care as contributing factors.

## Theme 2: patient concerns and fears

This theme encompassed the psychological and emotional dimensions of living with a hernia and making treatment decisions. Posts reflected a high degree of uncertainty, anxiety, and hesitation, particularly when surgery was presented as elective or precautionary.

### Fear of surgery and anaesthesia

Anxiety around being “*put to sleep*” was recurrent. Several posters asked others to “*talk me out of this surgery*,” fearing complications from anaesthesia more than the hernia itself. One user wrote, “*The idea of going under terrifies me more than the hernia. I just don’t trust it*.” This fear was often amplified by stories of unexpected adverse outcomes.

### Mesh-related anxiety

The topic of mesh was a powerful source of anxiety, even among those not yet offered surgery. Individuals recurrently used phrases like “*terrified of mesh*” or “*horror stories everywhere*.” Some contributors linked these concerns to decisions to defer surgery or seek alternative approaches, including treatment overseas. Even those who had not yet undergone surgery expressed a desire for reassurance: “*Will this mesh ruin my life in 10 years?*”

### Impact on daily life

Hernias affected basic functioning and lifestyle. Patients reported limitations such as being unable to carry groceries, care for children, or sleep comfortably. One user said, “*I haven’t played with my kids properly in months…it’s too painful.*” Another wrote that she was “*essentially housebound*,” unable to manage without assistance.

### Body image and self-perception

Multiple contributors expressed distress about their appearance. One post described the hernia as “*like carrying a basketball under my shirt*,” while others lamented persistent bulges or loose skin even after repair. Surgical scars, drains, and swelling also featured prominently, with one patient stating, “*Now I’ve got this huge scar*,* and it still looks weird*,* so was it worth it?*”

### Uncertainty and information seeking

Information gaps were recurrently reported, leading individuals to seek support from the group. Some questioned surgeons’ advice: “*He said it was elective*,* but then I read that hernias don’t go away and now I don’t know what to do*.” Others described getting second opinions or asking radiologists to re-read scans. The online group was described by some contributors as an important source of information during decision-making: “*I feel more informed here than I did after my consult.*”

## Theme 3: healthcare system and access to care

A prominent theme was the structural and systemic barriers to hernia care, particularly delays, inequities, and mistrust.

### Delays in diagnosis and treatment

Contributors across multiple threads recounted multi-year delays in diagnosis despite persistent symptoms. One user detailed: “*Three ultrasounds*,* five CTs*,* and three exploratory surgeries before they found the hernia*.” Others highlighted delays due to being misdiagnosed or told their symptoms were “*all in their head*.” Frustration and exhaustion were common responses.

### Insurance and financial barriers

In countries without universal coverage, insurance constraints strongly shaped options. One user explained, “*I’m saving up for Shouldice because I don’t trust mesh and insurance won’t pay for it*.” Others mentioned being denied revision surgery or mesh removal due to lack of coverage. Financial precarity affected both decision-making and recovery: “*I don’t get paid if I don’t work*,* so taking six weeks off is just not possible*.”

### Doctor–patient communication and trust

Mistrust in healthcare providers was evident across several discussions. “*My surgeon downplayed the risks*,” said one, while another described being “*brushed off*” when expressing mesh-related concerns. Contributors across multiple threads suggested that surgeons were incentivised to use mesh or lacked skill in alternative techniques.

### Weight management as a gatekeeper

Weight was, in several discussions, framed as a barrier to surgery. Contributors described being told to “*lose 30 pounds*” before being considered for repair, which many described as disheartening or unrealistic. One post read: “*It’s a catch-22*,* I can’t exercise properly because of the hernia*,* but they won’t fix it until I lose weight*.”

### Geographic disparities in care

Access to non-mesh surgery, high-volume surgeons, or specialist centres was described as varying considerably by location. One user sought surgery abroad due to local limitations, while others reported travelling hundreds of miles to consult a specific surgeon. Geographic inequity was a recurrent theme: “*I live in a small town where there’s no one here who even does these properly*.”

## Theme 4: coping strategies and support

Despite these challenges, multiple contributors described ways of adapting, connecting, and maintaining resilience.

### Online communities and peer support

Contributors frequently described the group as an important source of reassurance and peer support. Posts thanked members for “*getting me through this*” or “*calming me down*.” Contributors returned to share postoperative updates and offer reassurance to others. “*This group is better than Google*,” one remarked.

### Lifestyle modifications

People adapted their lives to accommodate their hernias. Strategies included avoiding lifting, using abdominal binders, and modifying diet. Some developed personalised systems: “*I’ve created a binder out of an old cloth and it works better than the hospital one.*” Others described “*reengineering*” their daily routines.

### Emotional resilience and hope

Alongside fear and frustration, posts also expressed hope and resilience. Posts celebrated small milestones: “*Today I walked to the mailbox pain-free!*” Others offered encouragement: “*If I can get through this*,* you can too.*” Some used humour or gratitude to reframe the experience.

### Advocacy and persistence

A final subtheme involved patients actively pushing for better care. Several reported self-advocacy leading to diagnosis: “*I made the radiologist reread the scan*,* and only then did they find it.*” Others travelled abroad, challenged their GPs, or pursued specialist care despite barriers.

## Cross-cutting observations across themes

Across all four themes, several higher-order observations emerged that extended beyond individual thematic categories. First, participants recurrently demonstrated active engagement in evaluating and negotiating treatment options, including discussion of operative approach, mesh use, surgeon selection, and interpretation of risk. These narratives suggested a form of patient-led surgical literacy and decision-making agency that extended beyond passive receipt of clinical advice.

Second, mesh-related discussions extended beyond immediate procedural considerations, and incorporated symbolic and emotional meanings. Contributors described concerns relating to toxicity, rejection, interaction with surrounding organs, permanence, and possible long-term effects. Alongside these negative accounts, other contributors described successful mesh repair and substantial improvement following surgery. Taken together, these discussions demonstrate that mesh was an emotionally salient topic, although the observational nature of the study limits inference regarding the deeper psychological meanings attached to these accounts.

Third, online interactions appeared to influence perceptions of risk and recovery through collective interpretation and validation. Participants recurrently drew upon peer narratives to contextualise their own experiences, seek reassurance, challenge recommendations, and evaluate future decisions.

Finally, contributors recurrently described how wider contextual factors including geography, body weight, financial circumstances, and perceived healthcare responsiveness shaped their experience of illness and treatment.

## Discussion

This study explored how individuals with hernia construct, negotiate, and communicate their experiences within a large online peer-support community. Four interconnected themes were identified, encompassing surgical experiences and outcomes, patient concerns and fears, healthcare access, and coping and support. Collectively, these findings reinforce the substantial biopsychosocial burden described in previous AWH research, while illustrating how treatment uncertainty, healthcare experiences, and peer interaction are articulated within an online environment. The findings are considered in relation to established quality-of-life research, including the York Model, alongside psychological and sociological perspectives on illness, online health information seeking, and the implications for patient-centred care.

The breadth of themes observed aligns with prior literature that positions hernia as a condition with significant biopsychosocial consequences [6], including impact on function [7], body image [4] and mental health [5]. However, our data demonstrate in granular detail how patients conceptualise and communicate these burdens, particularly in an online peer-to-peer setting. Surgical narratives recurrently extended beyond procedural outcomes and appeared to function as vehicles through which participants interpreted uncertainty, vulnerability, and trust in healthcare systems. Similarly, Patient Concerns and Fears encompassed anxiety around surgery and concerns regarding the bodily implications and long-term consequences of mesh implantation. Participants used vivid language, including descriptions such as being “stitched up with plastic” or “contaminated forever”, illustrating the emotional salience of mesh beyond its immediate technical role. The theme of Healthcare System and Access to Care highlighted structural and interpersonal obstacles to hernia treatment, including delayed diagnosis, geographic disparities, weight-based eligibility requirements, and perceived clinician dismissiveness. These experiences were sometimes associated with increased self-advocacy and pursuit of alternative opinions or treatment pathways. Online interactions also appeared to facilitate collective meaning-making, with resilience and reassurance emerging through repeated cycles of peer interpretation and support.

One possible framework through which to interpret these narratives is Leventhal’s Self-Regulation Model (SRM) of illness representation, which describes how individuals develop cognitive and emotional representations of illness and treatment that may influence coping and health behaviours [32, 33]. In this dataset, posts reflect distinct illness prototypes, where mesh, recurrence, or surgery itself are constructed as threats to physical and psychological integrity. These representations shaped emotional responses (e.g., anxiety, dread) and action plans (e.g., seeking non-mesh options, delaying surgery), reinforcing the model’s framework of identity, cause, timeline, consequences, and controllability.

In parallel, Symbolic Interactionism offers an interpretive lens through which the socially co-constructed meanings of surgical interventions and mesh could be understood. This sociological tradition emphasises how individuals derive meaning through interaction and symbolic language [34]. Metaphors such as “stitched up with plastic” or “caged” signal more than physical concerns, and they construct surgical mesh as a symbol of violated bodily boundaries and loss of agency. Within the digital setting, these meanings are continuously negotiated or resisted in dialogue with peers, illustrating how identity and illness narratives are socially constructed through interaction [35, 36].

The findings of this study closely resonate with established research on online health information seeking (OHIS), particularly in the context of contested conditions and high decisional conflict. Digital health consumers increasingly consult peer-led spaces prior to or in combination with professional advice, and this is particularly true of young users [10, 37]. Patients in this hernia support group sought social validation and shared experience, consistent with what Ziebland and Wyke (2012) have termed “narrative evidence” in the digital patient experience [38]. These narratives served as powerful alternatives to traditional clinical consultation, especially when patients perceived information gaps or conflicting advice in their medical care. Users exhibited characteristics described in the literature as “active information seekers”, displaying high agency in decision-making and often bypassing or challenging clinical hierarchies [39]. However, such digital health environments are also susceptible to the amplification of fear and misinformation, particularly when posts with strong emotive content (e.g., traumatic mesh failure stories) receive more engagement than nuanced or evidence-based discussions. The influence of emotionally charged testimony on risk perception is known [40, 41], and in this group, such narratives contributed to cycles of hesitancy and pursuit of alternative surgical strategies.

These findings can be compared to the York Model of Quality of Life in Abdominal Wall Hernia (AWH) as a sensitising framework developed through formal, qualitative interviews with CAWH patients [2]. This model, developed specifically for CAWH populations, includes five superordinate domains (Body Image [4], Mental Health [5], Symptoms and Function [7], Employment and Financial Impact, Interpersonal Relationships [6]) each with subordinate components. Broadly, there was concordance between our data and the five superordinate domains of the York Model.

Comparison with the York Model suggests broad conceptual alignment across established domains of mental health, body image, symptoms and function, financial burden, and interpersonal experience; however, the present findings indicate several areas where online patient discourse may extend existing conceptual boundaries. In particular, the prominence of surgical literacy and decisional agency suggests that patients actively construct treatment understanding through appraisal of operative approaches, surgeon selection, and perceived autonomy rather than acting as passive recipients of information. Similarly, while the York Model captures anxiety and coping, online narratives demonstrated that mesh recurrently acquired symbolic meanings beyond its technical role, being framed as a contaminant, permanent bodily alteration, or threat to identity, potentially intensifying emotional and body-image burden. The findings also suggest that contextual factors including geography, body weight, socioeconomic circumstance, and perceived healthcare responsiveness may operate as amplifiers of distress rather than simply determinants of access. Finally, the dynamics of online peer interaction appeared to extend beyond traditional support structures, with communal validation and collective risk appraisal shaping interpretation of illness, confidence in treatment decisions, and emotional resilience. Together, these observations support the York Model while suggesting opportunities for future refinement to better capture digitally mediated experiences of living with AWH. These observations should not be interpreted as validation of, or evidence for revision of, the York Model. Rather, they identify candidate concepts that may warrant further investigation through dedicated qualitative and psychometric research.

The thematic analysis of patient narratives within the online hernia support group presents an opportunity for healthcare providers to engage more proactively with patient information needs. Users recurrently turned to online spaces due to perceived informational voids or distrust in conventional healthcare encounters. In light of this, providers may wish to consider adopting a pre-emptive reassurance and education approach. This includes transparent communication around surgical risks (including mesh), managing expectations around recovery, and acknowledging common fears such as those surrounding anaesthesia or recurrence. Enhancing access to structured, patient-facing decision aids, co-designed with patient input, may represent one potential strategy to help mitigate the anxiety and uncertainty that fuels online information seeking [11].

The findings may also generate candidate areas for future research into patient-centred HRQoL assessment, including mesh-related concerns, postoperative functional uncertainty, healthcare trust, and digitally mediated support. Existing tools often insufficiently capture the lived psychosocial burdens reflected in this analysis [42]. By integrating patient-voiced constructs such as mesh-related fear, post-operative functional uncertainty, or distrust in care continuity, HRQoLs can move beyond generic symptom checklists and toward more contextually rich assessments taking account of the full breadth of lived experience of AWH. However, this exploratory study was not designed to assess the content validity or performance of existing PROMs, and therefore cannot establish that current measures require modification. Further targeted qualitative work and formal psychometric evaluation would be required to determine whether these concepts warrant representation within existing or future HRQoL measures.

## Limitations

This study was conducted solely through passive observation of an online support group that is hosted on Facebook and conducted in English-language. The group is endorsed by relevant stakeholders, including the European Hernia Society (EHS) [43]. However, given the community conducts itself in English-language it is important to note that cross-cultural issues and considerations may not have been captured. No other large online peer-communities conducting themselves in other languages could be identified. However, the European Hernia Society has recently introduced “chat rooms” in a variety of languages, and this may be a source of future exploration.

Passive observation meant that while minimising disruption to the community, there was limited opportunity to clarify meaning or delve deeper into participants’ intentions or experiences. A complementary study may choose to perform further active interaction with members in the form of “focus groups” or “interviews” to provide contextual richness. Furthermore, community demographic information (e.g. age, sex, comorbidities, socioeconomic background) was not collected and therefore no exploration into how experiences differed between or within demographic groups could be performed. The inability to reliably assign demographic or clinical characteristics to individual contributors limits interpretation of subgroup differences and reflects the methodological boundaries of netnographic approaches. Also, the study was not designed to quantify prevalence of opinions or experiences, and thematic prominence should not be interpreted as representative frequency.

Online support communities are self-selecting social environments and may overrepresent individuals with stronger motivations to seek support or share experiences. Although no overt evidence of systematic exclusion, moderation-driven suppression, or cyberbullying of alternative viewpoints was identified during data collection, subtle social influences and platform effects cannot be excluded. Findings should therefore be interpreted as analytically transferable rather than statistically generalisable.

A further limitation relates to the inability to verify the identity or motivations of individual posters. Although no overt legal, commercial, or solicitor-led recruitment content was identified during data extraction, covert participation by non-patient actors cannot be excluded. The findings should therefore be interpreted as analysis of online peer-support discourse rather than verified patient-level accounts.

It is noted that hernia type, anatomical complexity, and operative technique were not consistently specified by posters. As a result, the analysis cannot distinguish reliably between narratives relating to simple primary hernias, recurrent or incisional hernias, inguinal hernias, parastomal hernias, or complex abdominal wall reconstruction. The findings should therefore be interpreted as reflecting broad online discourse around hernia experience rather than technique-specific or diagnosis-specific outcomes.

Finally, although the vast majority of the group is made up of members of the public, there is a small minority of medical professionals as members, including surgeons. This minority is often called upon to answer specific questions and will largely limit their interaction to this, not interacting otherwise. However, it is possible that some members of the group may feel they are not able to fully express themselves as a result of this. In parallel, a number of members of the group specifically asked questions to “expert” surgeon members of the group, and therefore this aspect of OHIS directly to clinicians may be worth exploring in future studies.

## Conclusion

This netnographic study offers a unique account of how individuals affected by hernia navigate and share their lived experience in an online peer support community. The inductive themes (*Surgical Experiences and Outcomes*, *Patient Concerns and Fears*, *Healthcare System and Access*, and *Coping and Support*) demonstrate the complexity and heterogeneity of patient experience, extending well beyond symptom severity or anatomical classification. Mesh-related anxiety, fear of recurrence, mistrust in providers, challenges in navigating healthcare systems, and the psychological toll of disfigurement were recurrent motifs, rendered in a *chiaroscuro* of hope and despair.

These findings reinforce and extend the established York Model of Quality of Life in AWH [2], demonstrating strong convergence in key domains such as Mental Health and Symptoms and Function. However, there were identified potential areas for model refinement. In particular, the prominence of mesh-related ontologies and digital peer validation suggest the need for more granular and contextually attuned subdomains within patient-centred assessment frameworks. These insights carry implications for the development and refinement of HRQoL assessment tools for AWH. Conventional PROMs may insufficiently capture the complex, affective, and socially mediated burdens articulated in this analysis.

The study also highlights the broader implications of online health information seeking (OHIS) in the context of contested and high-decision-load surgical conditions. Patients demonstrated high agency, actively seeking narrative-based reassurance and social proof in the face of perceived clinical ambiguity or disempowerment. However, digital platforms introduce risk through the amplification of emotive testimony, which can skew risk perception and reinforce decisional paralysis. There is, therefore, an imperative for healthcare providers and service designers to proactively engage with patient information needs through transparent communication and co-produced decision aids. Clinicians should recognise the digital patient as an active information-seeker navigating a heteroglossia of clinical, experiential, and peer-generated voices within online communities. 
